# Optical Coherence Tomography Angiography of Macular Telangiectasia Type 2 with Associated Subretinal Neovascular Membrane

**DOI:** 10.1155/2017/8186134

**Published:** 2017-11-09

**Authors:** Victor M. Villegas, Jaclyn L. Kovach

**Affiliations:** Department of Ophthalmology, Bascom Palmer Eye Institute, University of Miami Miller School of Medicine, Miami, FL, USA

## Abstract

Optical coherence tomography angiography (OCTA) is a recently established noninvasive technology for evaluation of the retinal and choroidal vasculature. The literature regarding the findings in macular telangiectasia type 2 (MacTel2) is scarce. We report the OCTA findings associated with a subject with MacTel2 and secondary subretinal neovascularization (SNV). The commercially available Cirrus 5000 with AngioPlex (Zeiss, Jena, Germany) was used, without any subsequent image modification or processing. Subretinal neovascularization was detectable with OCTA at the level of the outer retina and choriocapillaris. Microvascular abnormalities associated with MacTel2 were present mostly in the deep capillary plexus of the retina temporally.

## 1. Introduction

Macular telangiectasia type 2 (MacTel2) is a bilateral retinovascular disease that is typically acquired during middle age and may lead to visual loss [1–3]. The hallmark of the disease is retinal vascular ectasia and neural atrophy of the macula [4]. This condition may pose a diagnostic challenge when evaluated with indirect ophthalmoscopy due to the subtle foveal findings. Initially, the only finding on fundus examination could be a decrease in retinal transparency temporal to the fovea. In the early nonproliferative phase of this condition, other clues to diagnosis can be an increase in central foveal autofluorescence related to a reduction in macular pigment. Fluorescein angiography typically demonstrates leakage from abnormal retinal vessels temporal to the fovea. Spectral domain optical coherence tomography (SDOCT) has offered great insight into the pathogenesis of this condition and shows atrophic abnormalities throughout the retinal layers in the foveal including an inner lamellar cyst with internal limiting membrane drape and disruption of the ellipsoid zone. These changes are thought to be due to degeneration of Müller cells and photoreceptors [5, 6]. Secondary subretinal neovascularization (SNV) can arise in the proliferative phase and form connections with choroidal vessels [7]. The presence of these vessels can be challenging to identify on FA in the setting of temporal foveal leakage.

Optical coherence tomography angiography (OCTA) is a new, fast, noninvasive imaging modality that allows detection of blood flow through the retinal and choroidal plexuses without intravenous dye injection [8]. Various retinal and choroidal diseases have been described using OCTA, including macular telangiectasia [9–20]. Currently, the literature regarding OCTA characteristics of MacTel2 patients with SRNV is limited.

The purpose of this report is to discuss the OCTA features of nonproliferative and proliferative MacTel2 imaged with OCTA. The commercially available Cirrus 5000 with AngioPlex (Zeiss, Jena, Germany) was used, without any subsequent image modification or processing.

## 2. Case Report

A 63-year-old male with history of well-controlled type 2 diabetes mellitus, essential hypertension, and heart disease was referred to our clinic due to visual loss in the right eye (OD). Prior ophthalmic history included radial keratotomy and LASIK in both eyes (OU). He also had uneventful cataract surgery with intraocular lens implantation OD and YAG capsulotomy. Best-corrected visual acuity was 20/50 OD and 20/20 in the left eye (OS). Manifest refraction was −1.00 + 1.50 × 52 OD and +1.50 + 1.75 × 42 OS. Intraocular pressure was 17 mmHg in both eyes (OU). Pupils were equally round and equally reactive to light. Anterior segment examination was remarkable for radial keratotomy with pseudophakia OD and nuclear sclerosis +1 OS. No evidence of intraocular inflammation was seen.

**Figure 1:**
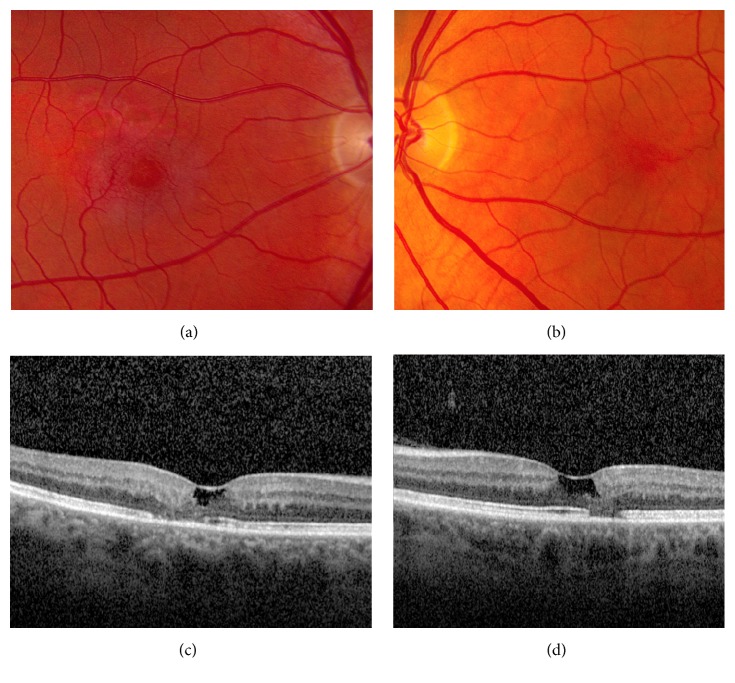
Macular telangiectasia type 2 initial presentation. (a) Fundus photograph of the right macula shows foveal pigment mottling and changes in the perifoveal vasculature. (b) Fundus photography of the left macula shows foveal pigment mottling. (c) Spectral domain optical coherence tomography (SDOCT) reveals an atrophic cyst and ellipsoid zone disruption in the right macula. (d) Similar SDOCT changes are present in the left macula.

**Figure 2:**
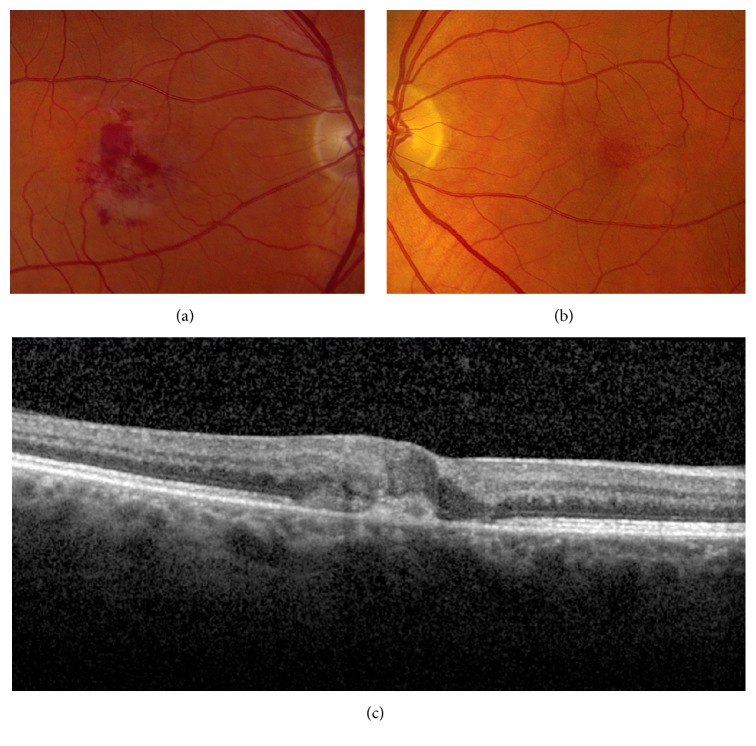
Fundus photography 2 years after initial presentation. (a) Macular hemorrhages are present in the right eye. (b) Stable foveal pigment mottling is present in the left eye. (c) SDOCT of the right macula reveals a subretinal neovascular membrane.

Fundus examination was remarkable for foveal pigment mottling OU. No diabetic or hypertensive retinopathy was present. SDOCT revealed atrophic cysts and ellipsoid zone disruption OU (Figure 1). Given the characteristic SDOCT findings, a diagnosis of macular telangiectasia type 2 was made and the patient was monitored every 6 months. Two years after initial examination, the patient developed acute visual loss to the level of 20/150 and presented with macular hemorrhage OD (Figure 2).

**Figure 3:**
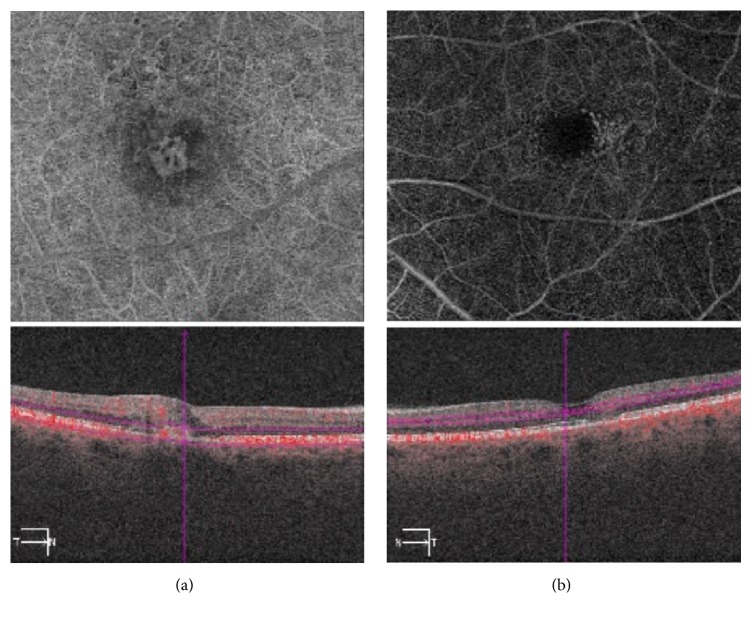
(a) OCTA OD at the level of the outer retina and choriocapillaris shows subretinal neovascularization. (b) OCTA OS demonstrates microvascular abnormalities in the deep capillary plexus of the retina most prominent temporally.

Macular OCTA was performed bilaterally. OCTA OD at the level of the outer retina and choriocapillaris shows subretinal neovascularization (Figure 3). OCTA OS reveals microvascular abnormalities in the deep capillary plexus of the retina most prominent temporally.

## 3. Discussion

This case demonstrates clinical features of nonproliferative and proliferative MacTel2 imaged with OCTA. Early in this condition changes in the retinal microvasculature begin temporal to the fovea in the deep capillary plexus. These abnormalities then extend circumferentially and into the superficial capillary plexus. Anastomoses form between both plexuses and retinal atrophy progresses. This can progress to SRN that can form connections with choroidal vessels [21].

OCTA technology can facilitate diagnosis of all stages of MacTel2 and essentially obviate the need for FA which does not discriminate between superficial and deep retinal vasculature. Also, the detection of subretinal neovascularization in cases in which there is no macular hemorrhage and when temporal leakage on FA is prominent can become challenging on FA.

MacTel2 etiology continues to be poorly understood. Perifoveal Müller abnormalities may be a common pathway associated with the disease [1, 4]. Loss of regulation of these cells may lead to photoreceptor death, dysregulation of angiogenic and inflammatory factors, vascular ectasia, and subsequent subretinal neovascularization [22]. Histopathologic studies have shown thickening of retinal capillaries and loss of Müller cells in subjects with macular telangiectasia [6, 23]. Recent studies have suggested that subjects with diabetes mellitus and hypertension are more likely to have MacTel2 [24]. This may be due to ischemic changes at the level of Müller cells. However, the mechanisms behind systemic disease and MacTel2 remain poorly understood. Further studies will elucidate the mechanisms that explain such associations.

In conclusion, OCTA is a new, fast, noninvasive imaging technology that has enabled improved understanding of the pathophysiology of many retinal vascular diseases including MacTel2. For this condition, OCTA not only facilitates diagnosis but also enables the clinician to monitor disease progression and quantify response to anti-VEGF therapy. Future studies with OCTA will hopefully illuminate additional features of MacTel2 and provide a better understanding of this complicated disease.
